# The Functional Versatility of Transferrin Receptor 2 and Its Therapeutic Value

**DOI:** 10.3390/ph11040115

**Published:** 2018-10-23

**Authors:** Antonella Roetto, Mariarosa Mezzanotte, Rosa Maria Pellegrino

**Affiliations:** Department of Clinical and Biological Sciences, University of Torino, 10043 Orbassano, Torino, Italy; mariarosa.mezzanotte@unito.it (M.M.); rosamaria.pellegrino@unito.it (R.M.P.)

**Keywords:** Tfr2, iron metabolism, hepcidin, erythropoiesis, SNC

## Abstract

Iron homeostasis is a tightly regulated process in all living organisms because this metal is essential for cellular metabolism, but could be extremely toxic when present in excess. In mammals, there is a complex pathway devoted to iron regulation, whose key protein is hepcidin (Hepc), which is a powerful iron absorption inhibitor mainly produced by the liver. Transferrin receptor 2 (Tfr2) is one of the hepcidin regulators, and mutations in *TFR2* gene are responsible for type 3 hereditary hemochromatosis (HFE3), a genetically heterogeneous disease characterized by systemic iron overload. It has been recently pointed out that Hepc production and iron regulation could be exerted also in tissues other than liver, and that Tfr2 has an extrahepatic role in iron metabolism as well. This review summarizes all the most recent data on Tfr2 extrahepatic role, taking into account the putative distinct roles of the two main Tfr2 isoforms, Tfr2α and Tfr2β. Representing Hepc modulation an effective approach to correct iron balance impairment in common human diseases, and with Tfr2 being one of its regulators, it would be worthwhile to envisage Tfr2 as a therapeutic target.

## 1. Tfr2 Gene and Proteins

Tfr2 is a type II transmembrane glycoprotein, a member of the transferrin receptor family and homologous to Tfr1 [1].

**Figure 1:**
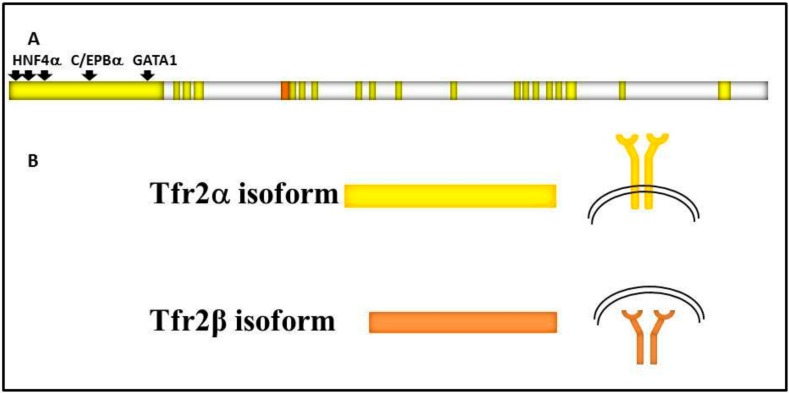
Schematic representation of: (**A**) *TFR2* gene structure. *TFR2* 18 exons are in bright yellow, *TFR2α* promoter is shown upstream of the gene, and transcriptional factors binding sites are highlighted by black arrows. 5′ untranslated region (142 bp long) of *TFR2β* transcript is shown in orange; (**B**) the two main transcripts and of Tfr2α and Tfr2β isoforms, that are identical in the common sequence, and the two protein localizations, on the cell surface or in the cytosol, respectively.

It is encoded by *TFR2*, a 2471 bp long gene localized on the long arm of human chromosome 7 (7q22.1) that consists of 18 exons, and gives origin to two main variants regulated by different specific promoters: Tfr2α and Tfr2β (Figure 1).

Tfr2α results from the transcription of all exons, and is prevalently and highly expressed in hepatocytes and erythroid cell lines. Tfr2α cDNA is 2.3 kb long (AF067864), and the Tfr2α is a protein of about 89 kDa encompassing 801 amino acids [2]. As Tfr1, Tfr2α has a short cytoplasmic tail (aa 1–80) that contains a consensus sequence YQRV for endocytosis, a transmembrane domain (aa 81–104) with four cysteines (aa 89–98 and 108–111), involved in disulphide bonds, likely responsible for *TFR2* homodimerization, and a large extracellular domain (aa 105–801) comprising a protease-associated domain and two RGD motifs that bind di-ferric Tf (Fe2Tf). Furthermore, an N-terminal mitochondrial targeting sequence (MTS) has been found in Tfr2 intracellular domain [3]. In vitro analysis demonstrated that Tfr2α on cell membranes can be shed and give origin to a soluble form, and that this process is inhibited by Fe2Tf [4]; however, this form could not be found in animal or human sera.

Tfr2α transcription is upregulated in mouse embryonic fibroblast cells (NIH3T3) by erythroid GATA1, EKLF, and cEBP/α transcriptional factors, while FOG1 seems to inhibit GATA1 enhancement [5]. Also, hepatic Hnf4α stimulates Tfr2α transcription, since it is significantly decreased in liver-specific HNF4α-null mice [6]. There is no Tfr2α IRE/IRP-dependent post-transcriptional regulation [7], while the hepatic tetraspanin CD81 is able to interact with Tfr2α and induce its degradation [8].

Tfr2β has an in-frame transcription start site in exon 4, so the Tfr2β cDNA (NM_001206855.1) transcript lacks exons 1–3, and presents 142 additional untranslated base pairs at its 5′ end. Tfr2β is ubiquitously expressed at low level, and mostly expressed in spleen, heart, and brain. The resulting protein lacks the cytoplasmic and the transmembrane domain [2]. Since no signal peptide involved in the secretory pathway could be evidenced in Tfr2β isoform, it is supposed to be a cytosolic 60 kDa protein identical to the Tfr2α extracellular domain. At the moment, no transcriptional/translational regulatory pathway is known for Tfr2β isoform (Figure 1).

### 1.1. Tfr2 and HFE3

Inactivating mutations of *TFR2* gene (OMIM: 604720) lead to type 3 hereditary hemochromatosis (*TFR2*-HHC or HFE3), a rare recessive disorder characterized by increased transferrin saturation and serum ferritin concentration and iron overload [9].

HFE3 is one of the 5 different forms of hereditary hemochromatosis, a genetically heterogeneous disorder due to the deregulation of iron protein hepcidin (Hepc) [10] (Table 1). *TFR2*-HHC presents an earlier age of onset than type 1 hereditary hemochromatosis (*HFE-HHC*), and some pediatric patients have been reported so far. However, the majority of the affected individuals are young adults with abnormal serum iron indices [11]. 

Most of the mutations involved in HFE3 pathogenesis cause an inactivation of both Tfr2 isoforms, but some of them, occurring in exons from 1 to 3, impair the production of the Tfr2α isoform only [12,13,14]. Three patients with homozygote mutation M172K, that impairs Tfr2β translation initiation codon, were identified, all presenting typical hemochromatosis symptoms (cirrhosis, hypogonadism, cardiomyopathy, arthritis) at an average age of 38 ± 5 years [13,15].

Unfortunately, few clinical data are available on patients with these mutations to allow an exhaustive genotype/phenotype analysis.

### 1.2. Systemic Iron Metabolism: The Hepc-Fpn1 Axis and the Proteins Involved in Hepc Regulation

In mammals, the hepatocyte-secreted hormone Hepc regulates systemic iron homeostasis [16]. Hepc is codified by *HAMP* gene, which encodes for an 84 amino acids precursor protein, from which active 20–25 amino acids peptides are generated [17]. It is expressed primarily in the liver, although low levels of Hepc transcripts have been also reported in other organs [18].

How *HAMP* gene expression is regulated is mostly unknown. There are no IRE elements in its transcript, but the transcriptional factor CCAAT/enhancer binding protein-α is highly expressed in the liver, and seems to stimulate *HAMP* expression, while the hepatocyte nuclear factor 4-α (HNF-4) represses Hepc expression [19].

The molecular processes involved in hepatic Hepc regulation are quite complex. Basal Hepc expression is regulated through the bone morphogenetic protein 6 (Bmp6) and Smad protein signaling pathway. In iron excess condition, Bmp6, produced and secreted by liver sinusoidal endothelial cells (LSECs) [20], binds to bone morphogenetic protein receptors, ALK2 and/or ALK3 [21], activin receptor type 2A (Actr2a) [22], Hemojuvelin (Hjv) and Neogenin [23]. The protein complex activates signals transducers Smad1/5/8, leading to their interaction with the common mediator Smad4. As a consequence of this interplay, Smad4 translocates into the nucleus and promotes Hepc transcription [16].

More recently, it has been demonstrated that bone morphogenetic protein 2 (Bmp2), expressed in LSECs, can also trigger Hepc transcription increase [24]. 

A second Hepc regulatory pathway involves di-ferric Tf (Fe2Tf) as the signaling of increased iron availability, transferrin receptor 1 (Tfr1), hemochromatosis type 1 protein (Hfe), and transferrin receptor 2 (Tfr2). It has been demonstrated that Fe2Tf competes with Hfe for binding Tfr1 then, when circulating, Fe2Tf increases as a consequence of iron raising, Hfe dissociates from Tfr1 and binds Tfr2 [25]. Hfe/Tfr2 complex is then responsible for Hepc response to iron increase, through the activation of Erk1/2 and MAPK cascade that has been proposed to potentially converge on the Bmps/Smad1/5/8-mediated pathway [26].

**Figure 2:**
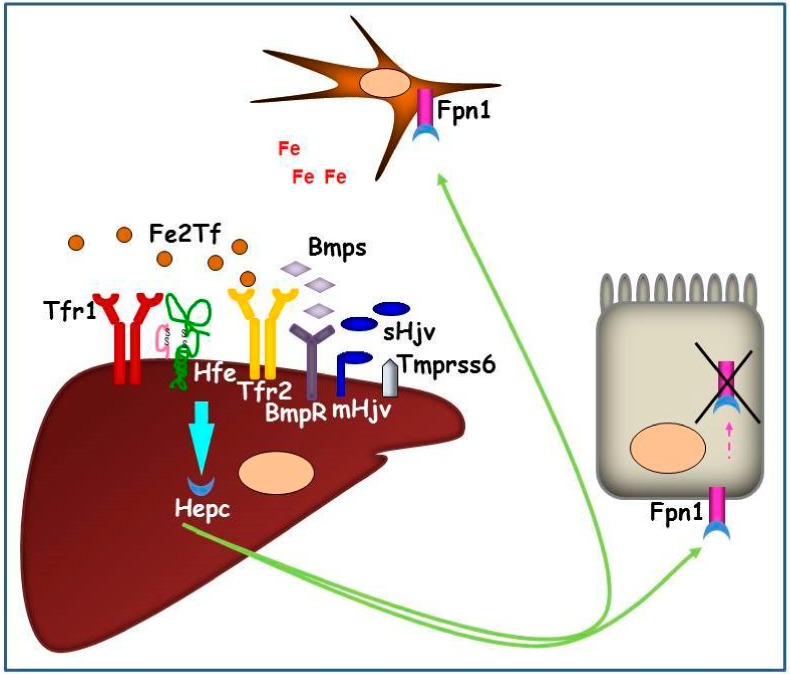
Graphic representation of the hepatic Hepc pathway in conditions of systemic iron increase. The iron signaling proteins (Fe2Tf and Bmps) interact with iron sensors (Tfr1, Hfe, Tfr2, BmpRs) and their co-activator (mHjv) to promote Hepc production. Hepc, secreted by hepatocytes, is transported in plasma and binds iron exporter Fpn1 on duodenal and reticuloendothelial cell surfaces causing its internalization and intracellular degradation. As a consequence, iron remains entrapped in these cells, systemically reducing the metal availability.

The hierarchy of the two pathway activations, and their relationship, are still not completely defined. In vitro data support the hypothesis that the complex Hfe/Tfr2 interacts with membrane Hjv (mHjv) on cell surface, thereby, the link between the two signaling pathways occurs [27]. It has been found, in vivo, that both Hfe and Tfr2 knock-out (KO) mice present lower pErk1/2 [28] and pSmad1/5/8 proteins [29,30], meaning that these two proteins regulate both signal translation pathways. Hepatic Hepc upregulation is inhibited by matriptase 2 (MT-2 or Tmprss6), that acts as Hepc inhibitor cleaving mHjv expressed on the plasma membrane [31]. *TMPRSS6* gene expression has been found to be induced by chronic dietary iron loading and Bmp6 injection [32], and its interaction with Neogenin facilitates mHjv cleavage and inactivation in transfected cells [23] (Figure 2).

On the contrary, in iron deficient conditions, this signaling pathway is inhibited by soluble Hjv (sHjv) and Tmprss6, which physically interacts with mHjv, causing its fragmentation.

Hepc expression in hepatocytes is systemically regulated by multiple signals: body iron availability, such as iron-loaded transferrin and hepatic iron stores, erythropoietic activity, hypoxia, and inflammation [33]. Hepc secreted by hepatocytes regulates iron release from duodenal enterocytes, splenic macrophages, and hepatocytes, which are responsible for dietary iron absorption, contain large amounts of iron from erythrocyte recycling, and act as an iron reservoir and export iron when needed, respectively. Hepc exerts its function, binding the iron exporter ferroportin 1 (Fpn1) [34] and stimulating complex internalization and degradation, leading, de facto, to cellular retention of iron [35]. Elevated plasma Hepc, as in inflammatory state, downregulates iron efflux from several cell types, and this leads to an overall reduction in plasma iron. On the contrary, low Hepc, as seen in iron-depleted or erythropoietic expansion conditions, causes an increased iron release by macrophages and by the basolateral site of villi duodenal cells.

A potent Hepc inhibitor signal is iron demand for erythropoiesis, mediated by three Hepc modulators (Gdf15, Twgs1, Erfe). Their roles and precise mechanisms in Hepc regulation are still not completely clear, but Erfe, in particular, has emerged as a potent Hepc negative regulator in conditions of acute erythropoietic demand, acting in conjunction with erythropoietin (Epo) signaling, as well as in anemia of inflammation (AI) condition [36].

*HAMP* expression is induced by inflammation and infection. This acute phase response involves a different pathway from the ones described above, and is mainly mediated by interleukin 6 (IL-6) inflammatory cytokine, and requires the signal transducer and activator of transcription 3 (STAT3) activation, and the binding of STAT3 to a STAT3-binding motif in the Hepc promoter [37]. In addition, cytokine IL-22, involved in immunological response to extracellular infections, as well as Toll-like receptor 5 (TLR5) agonist flagellin, seem to upregulate Hepc, strengthening the hypothesis of a possible Hepc role in innate immunity [38].

Conversely, *HAMP* expression is repressed by hypoxia both in vitro and in vivo in animal models [33] and humans [39,40]. The mediator of Hepc response to hypoxia seems to be the hypoxia inducible factor (HIF), even though it is not clear if it acts directly or indirectly on Hepc regulation [16]. The fact that Hepc inhibitor, Tmprss6, presents a hypoxia responsive element (HRE) in its promoter [41] might make Tmprss6 the linking protein between hypoxia and iron homeostasis.

Mutations in *HAMP* gene and in genes codifying for Hepc regulatory proteins (*HFE*, *TFR2*, and *HJV*) cause the lack of upregulation of Hepc as a response to increased liver iron stores. In fact, inappropriately low levels of liver Hepc are observed in patients and mouse models of hereditary hemochromatosis [42,43]. These conditions promote a continuous dietary iron absorption that leads to iron overload. On the contrary, inappropriately high Hepc has been found in animal models and patients with iron resistant iron deficient anemia (IRIDA), a genetic disorder due to mutations in *TMPRSS6* gene [44,45,46]. 

Mutations in *HAMP* gene and in genes codifying for Hepc regulatory proteins (*HFE*, *TFR2*, and *HJV*) cause the lack of upregulation of Hepc as a response to increased liver iron stores. In fact, inappropriately low levels of liver Hepc are observed in patients and mouse models of hereditary hemochromatosis [42,43]. These conditions promote a continuous dietary iron absorption that leads to iron overload. On the contrary, inappropriately high Hepc has been found in animal models and patients with iron resistant iron deficient anemia (IRIDA), a genetic disorder due to mutations in *TMPRSS6* gene [44,45,46]. 

## 2. Tfr2 in Liver

In the liver, Tfr2α is a sensor of circulating iron, but the knowledge about the Tfr2α hepatic function is still incomplete. It is known that Tfr2α localizes in caveolar microdomains [47], membrane structures involved in the recruitment of receptors that can be activated by ligand binding [48]. Also, Tfr2α localizes in lipid raft domains on the exosomal cell membrane, where it is internalized by clathrin-mediated endocytosis, if transferrin saturation (TS) is low [49]. 

Tfr2α protein regulation occurs mainly through its stabilization on the cell membrane as a consequence of the binding to Fe2Tf [50,51]. An in vitro study showed that, in the presence of Fe2Tf, Tfr2α has an increased half-life and is recycled, while in presence of apo-Tf membrane, Tfr2α is mainly subjected to lysosomal degradation [52]. It has been recently demonstrated that CD81 is also able to induce Tfr2α degradation, but the correlation between this Tfr2α regulatory route and Hepc pathway is still obscure [8].

Therefore, increased TS has an opposite effect on the two Tfrs via two different mechanisms: it causes a decrease of Tfr1, regulated by the IRE/IRP system, but a stabilization of Tfr2α on the cell surface [1].

This supports the hypothesis that Tfr2α exerts its function(s) as a signaling receptor more than as an iron importer.

According to the available in vitro data, hepatic Tfr2α interacts, on the cell membrane, with two main iron proteins, Tfr1 and Hfe.

The current model assumes that Tfr2α, in conjunction with HFE and Tfr1, is a partner of a sensor complex of circulating iron that activates Hepc in response to elevated TS [53]. In physiological conditions (TS 30–35%) Hfe and the complex Tf/Tfr1 are bound on the plasma cells; when TS increases in response to increased iron availability, loaded Tf impairs Hfe binding to Tfr1/Tf complex, leading it to bind Tfr2α, that is stabilized on the membrane by the same Fe^2^Tf. The resulting complex Tfr2α/Fe2Tf/Hfe causes the activation of Hepc transcription [25]. On the HuH7 hepatoma cell surface, this Tfr2α/HFE interaction occurs within a multiprotein complex, that also includes mHjv [27]. It remains to be demonstrated if this complex activates the intracellular signaling to upregulate Hepc expression, also, in vivo. 

In the presence of Fe2Tf, Tfr2α is able to activate Erk1/2 and p38 MAPK kinase signaling transduction pathway [47], since Tfr2 KO mice present a decrease of pErk1/2 [28]. Furthermore, the Smad1/5/8 pathway also seems to be involved in Tfr2α-mediated signal transduction, since pSmad1/5/8 is decreased in Tfr2 KO animals, as well [29]. 

Erk 1/2 phosphorylation could be increased also by Hfe overexpression, and both Tfr2 and Hfe cause an increase of the pro-hormone convertase furin [29], previously demonstrated to be involved in Hepc regulation [54]. Whether it is the sole and/or the main Tfr2 dependent Hepc regulatory pathway is still not clear.

## 3. TFR2 Mouse Models

The first Tfr2 KO animal model was generated by targeted mutagenesis, introducing a premature stop codon (Y245X) in the murine Tfr2 coding sequence [55]. This mutation is homologous to the Y250X variant, originally detected in humans and responsible for HFE3 [9]. Even young homozygous Y245X mice maintained on a standard diet had high liver iron concentration, in agreement with the observation of early iron overload in HFE3 patients [11]. As in humans, heterozygous animals were normal. The histological distribution of iron resembles the features of HFE3, with the typical liver periportal accumulation. 

Subsequently, different murine models of Tfr2 inactivation were developed, including Tfr2 total (Tfr2 KO) and liver-specific (Tfr2 LCKO) knockouts [56,57] as well as a Tfr2/Hfe double KO [28]. All these models are characterized by an inadequate hepatic Hepc expression and liver iron overload with variable severity. However, when generated in the same genetic background, Tfr2 KO mice were shown to have a more severe iron overload than Hfe KO, although less severe than the Tfr2/Hfe double KO [58]. These observations are in agreement with the model of Tfr2/Hfe proteins’ cooperation in the liver. 

In a double Tfr2/Hjv KO mouse model, plasma Hepc and Hepc transcription was lower than in Tfr2, and similar to Hjv single KOs, respectively. The same was true for the Tfr2/Hfe double KO [59]. Also, a recent study on a mouse model with inactivation of both Bmp6 and Tfr2 (Tfr2/Bmp6 double KO) demonstrated that loss of functional Tfr2 further represses Hamp expression, Smad5 phosphorylation, and plasma Hepc amount in Bmp6 KO mice. The same results were obtained in the Hfe/Bmp6 double KO, and the Hfe/Bmp6/Tfr2 triple KO [60]. All these data support the hypothesis that Tfr2 and Hfe act downstream Bmp6 and upstream Hjv in Hepc regulatory pathway.

Last, Tfr2 germinal vs liver-specific KO animals highlighted a distinct function of Tfr2 outside the liver in maintaining iron balance. In fact, Tfr2 KO mice have less severe iron overload, slightly higher hemoglobin (Hb) levels [57,61], and moderate macrocytosis than Tfr2 LCKO [56,57]. 

To study the specific function of Tfr2β isoform in iron metabolism, a specific mouse model was generated, introducing the M167K substitution in the Tfr2 protein [57]. This mutation, homologous to the one found in naturally mutant individuals with HFE3, substitutes the start codon methionine of the Tfr2β isoform, with a lysine. Interestingly, this knock-in mouse model (Tfr2 KI), specifically lacking the Tfr2β-isoform (α^+^β^0^), is characterized by normal transferrin saturation, liver iron concentration, Hepc, and Bmp6 levels, but shows transient anemia at a young age. In addition, adult Tfr2 KI animals accumulate iron in the spleen, due to a significant reduction of iron exporter Fpn1 mRNA, thus suggesting a possible regulatory effect of Tfr2β isoform on splenic Fpn1 expression. These data are further supported by the results obtained in Tfr2 macrophage-specific KO mouse model. These animals present normal systemic iron parameters, but lower Fpn1 transcript and protein in peritoneal macrophages [62]. Recent studies demonstrated that Tfr2β is well expressed in reticuloendothelial cells of different tissues, where it exerts its role in modulating iron availability in these tissues, acting on Fpn1 transcription (see below). Since Fpn1 protein has several regulatory systems both at the transcriptional [63,64] and post-transcriptional level through IRE/IRP system [7], and origins from different Fpn1 transcripts with or without IREs [65], it remains to be clarified how and when Tfr2β acts on Fpn1 regulation. 

## 4. Tfr2 in Extrahepatic Tissues

### 4.1. Tfr2 in the Erythropoietic Compartment

A Tfr2α erythropoietic role was firstly hypothesized in genome-wide association studies that identified Tfr2α polymorphisms affecting hematologic parameters [66,67]. These data were further strengthened by the identification of Tfr2α as a component of the erythropoietin receptor (EpoR) complex in erythroid progenitor cells. Tfr2α was shown to be crucial for efficient transport of EpoR to the cell surface and for its terminal differentiation, since human erythroid progenitors with silenced Tfr2α showed a delayed differentiation [68]. Another hint was provided by the increased Hb content present only in Tfr2 germinal KO, but not in liver-specific KO mice. Since both mouse models manifest comparable iron overload, the lack of enhanced hemoglobinization in Tfr2 LCKO mice suggests that the erythroid function of Tfr2α is preserved [68]. Also, double Tmprss6/Tfr2 KO mice develop erythrocytosis while, in double Tmprss6/Tfr2 LCKO mice, where Tfr2α is functional in erythroid cells, red blood cells (RBC) number is normal [61].

Recently, a mouse model lacking Tfr2 in bone marrow cells (Tfr2^BMKO^) was developed injecting BM cells from Tfr2 KO mice in lethally irradiated C57/BL6 mice. Tfr2^BMKO^ mice manifest reduced mean corpuscular value (MCV) and low Hepc levels as a typical response to iron deficiency, but an enhanced terminal erythropoiesis, demonstrated by increased RBC and Hb content [69]. Interestingly, erythropoiesis and Epo level in these mice do not change in a mild dietary restriction setting, as happens for WT animals, where the Epo level is drastically increased.

As a whole, these data suggest that the lack of Tfr2 confers increased Epo sensitivity to erythroid progenitor cells, a hypothesis that is further supported by the induction of Epo target genes, like Hamp regulator Erfe [70], in these animals.

A similar animal model was recently developed crossing Tfr2 floxed mice with Vav-Cre expressing mice to obtain Tfr2 silencing in erythroid compartment [62]. Results differed from previous work since decreased RBC and splenomegaly were observed, but these discrepancies might be explained by the different procedures used to create the two mouse models since, in the first case, Tfr2 is silenced in all bone marrow (BM) cell lines after a BM transplant procedure while, in the latter, only the erythroid cell lines are Tfr2 null.

In another study, Tfr2 erythropoietic role was further investigated studying the erythropoiesis of two Tfr2 mice with one or both Tfr2 isoforms silenced (Tfr2 KI and Tfr2 KO), and with normal or increased iron availability [57]. The evaluations were performed in bone marrow and spleen, in young and adult animals to unravel the erythropoietic role of Tfr2 isoforms at different ages, and in the two main erythropoietic organs. It resulted that the lack of Tfr2 in Tfr2 KO mice leads to macrocytosis with low reticulocyte number and increased Hb value, together with an anticipation of erythropoiesis in young mice both in BM and in the spleen [71], probably because the increased systemic iron amount present in these animals allows them to reach mature erythropoiesis even at a young age.

Although different animals and approaches were used in these studies, and partially contradictory results were obtained, they all demonstrate that erythropoiesis is impaired by a lack of Tfr2 in BM, independently from its activity in hepatic tissues.

Moreover, results obtained studying Tfr2 KI (α^+^β^0^) mice [57] demonstrated, for the first time, the involvement of Tfr2β in favoring iron availability for erythropoiesis. In fact, the sole lack of Tfr2β, in normal systemic iron condition, causes an increased but immature splenic erythropoiesis seen only in young mice, as if they had insufficient iron availability during animal growth, that is normalized in animal adult age. Decreased iron availability for erythropoiesis in Tfr2 KI young mice is demonstrated by increased ferritin (Ft) and decreased divalent metals transporter 1 (DMT1) in their splenic monocyte, the increase of Erfe transcription in BM and spleen, and the low hepatic Hepc transcription that could, in turn, be responsible for the increased splenic Fpn1 amount in these animals [71].

**Figure 3:**
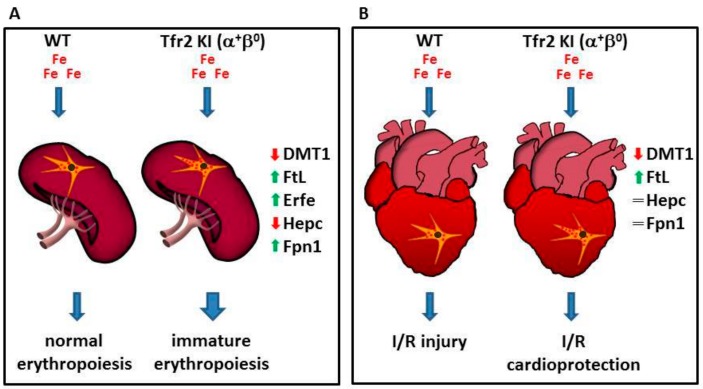
Schematic model illustrating Tfr2β function in regulation iron export from reticulo-endothelial (REL) cells. Lack of Tfr2β causes an increased iron retention in REL cells that (**A**) causes the onset of an immature erythropoiesis in the spleen, and (**B**) induces a cardioprotection against the effect of the reperfusion of oxygenated solutions after an ischemic event in heart (see text).

This effect, due to Tfr2β absence, in aged matched Tfr2 KO (α^0^β^0^) mice, was compensated by the increased amount of circulating iron available that may be used for erythrocyte production (Figure 3A).

### 4.2. Tfr2β in the Heart

The cardiac muscle is a major site of oxygen consumption, so an adequate intracellular iron pool is essential to its aerobic activity. This is demonstrated by the finding that deletion of cardiac Tfr1 in mice causes fatal energetic failure in cardiomyocytes [72]. Cardiomyocytes express relatively high levels of Hepc and Fpn1, despite the fact that these cells have no role in systemic iron control [73]. 

Studies on mouse models demonstrated that the cardiac Hepc/Fpn1 axis is essential for heart cells’ autonomous control of the intracellular iron pool that guarantees a normal cardiac functionality [73], and that Hepc/Fpn1 appears to protect the heart from the effects of systemic iron deficiency [74]. 

On the other side, cardiomyocytes are particularly susceptible to ROS-mediated damage because they are rich in mitochondria and consume large amounts of oxygen [75]. Therefore, when labile iron pool (LIP) expansion occurs, oxidative stress can affect cardiac functions, as it happens in severe juvenile HHC forms [10]. 

Although ubiquitously expressed, Tfr2β is highly transcribed in heart [2], such that a role for Tfr2β isoform in cardiac iron management has been postulated. 

Indeed, in the hearts of two Tfr2β null mice with normal or increased systemic iron amount, Tfr2 KI and Tfr2 LCKO [57], the silencing of Tfr2β induces a selective activation of different proteins involved in cell survival, antioxidant enzymes, and kinases involved in cardioprotective pathways that are usually activated by stressful stimuli.

In particular, Tfr2 KI and Tfr2 LCKO mice develop a greater resistance against acute ischemia/reperfusion (I/R) challenge, irrespective of animals’ systemic iron content, via the activation of the RISK or SAFE/GSK3β cardioprotective pathways, respectively. The iron imbalance present in these mice hearts was demonstrated by the finding that both models present the activation of antioxidant proteins, pro-apoptotic markers, and catalase, even before I/R [76]. They also have a slightly increased synthesis of cardiac ferritins, similarly to what happens in ischemic preconditioning, in which a small increase of ferritin protects cardiac cells from iron-mediated oxidative damage associated with ischemia/reperfusion injury [77].

Since previous data demonstrated a significant decrease of Fpn1, and an increased iron deposit in splenic macrophages in Tfr2β-null mice [57,71], one might hypothesize that Tfr2β isoform inactivation, in the heart, causes an iron retention in cardiac reticuloendothelial cells that is able to induce cardioprotective pathways activation and to reduce iron availability to form free oxygen radicals during the reperfusion phase (Figure 3B).

### 4.3. Tfr2 in the Central Nervous System (CNS)

Iron levels in the brain vary during life. The iron amount increases with aging in the striatum and the brain stem [78] and it is present in most CNS cell types: neurons, oligodendrocytes, microglia, and astrocytes [79]. A well-regulated iron homeostasis is important for brain development and function. Iron deficiency negatively impacts neurodevelopmental processes [80], and is also implicated in a number of psychiatric and neurological conditions, learning disabilities, attention deficit hyperactivity disorder (ADHD), and pediatric restless legs syndrome (RLS) [81,82]. On the contrary, brain iron overload is present in Alzheimer’s and Huntington’s neurodegenerative disorders, as well as in Parkinson’s disease (PD) [83]. Nevertheless, the exact role of iron in these diseases’ onset/worsening is still debated, and it remains to be clarified whether brain iron overload is directly involved in their pathogenesis, or it is a secondary effect that contributes to their clinical symptoms’ progression.

The main sites of brain iron uptake are the brain vascular endothelial cells (BVECs) present in the blood–brain barrier (BBB) [84]. As in other organs, there are two main pathways responsible for CNS cells’ iron uptake, the Tf-Tfr1 pathway, and the NTBI transport pathway. Traditionally, Tf-Tfr1 is considered a major pathway, and works as in all the other cell types of the organism, through a receptor-mediated endocytosis of plasma Tf circulating in the ventricles [85].

The NTBI transport pathway has been recently revaluated as a significant way to introduce iron in CNS, and it could be done through vesicular or non-vesicular mechanism. In the first case, Tf homologues, such as lactoferrin and melanotransferrin, might be involved in Tfr-mediated iron transport; moreover, the newly characterized Ft receptors, Tim 2 and Scara5, can introduce iron inside the cells through a Ft-FtR pathway. Non-vesicular iron uptake can be exerted by iron importer DMT1, that is present in endothelial cells of the brain microvasculature, as well as other importers like IN4/5/6 [86].

In CNS, the iron exporter Fpn1 is found in BVECs, neurons, oligodendrocytes, astrocytes, the choroid plexus, and ependymal cells and microglia, together with ceruloplasmin (CP) or hephaestin (Hp), the two ferroxidases that cooperate with Fpn1 to facilitate iron export [87]. Fpn1 could be the main protein responsible for iron release from CNS cells, even if other proteins and mechanisms have been brought into play for these processes [86].

Inside brain cells, the majority of iron is bound to ferritin heteropolymers (Ft H/L) [88]. Their cellular distribution and ratio varies with iron status, age, and disease conditions [89].

CNS iron homeostasis is intracellularly modulated by (IRE/IRPs) system [90], and by local and systemic Hepc. Injection of Hepc into the mouse lateral cerebral ventricle decreases Fpn1 protein levels and treatment of primary cultured rat neurons with Hepc decreases Fpn1 expression and reduces these cells release of iron [91]. More recently, it was demonstrated that injection of adenovirus expressing Hepc (ad-hepcidin) in brain ventricles reduces brain iron in iron-overloaded rats through the downregulation of iron transporter [92]. This data indicates that Hepc/Fpn1 axis is present and acts in CNS, as in the other districts of the organism.

It remains a matter of debate whether Hepc acting in brain is locally produced or comes from the systemic circulation crossing the BBB or both [93].

Similar to other Hepc regulatory proteins, Tfr2 gene expression has been shown in total brain extracts [2,94], in brain tumor cell lines [95], or in specific neuronal subtypes as dopaminergic neurons [3]. Furthermore, a transcriptome study on Tfr2-null mice revealed that several genes involved in the control of neuronal functions are abnormally transcribed [96]. Of note, the same experimental approaches, applied to Hfe-null mice, revealed that a consistent percentage of transcripts are modified in the same way in the two models [96]. This highlights the possibility of a cooperation between Tfr2α and Hfe protein in CNS iron regulation, as in the rest of the organism.

Immunofluorescence studies using a Tfr2α-specific antibody demonstrate that the protein is significantly produced in mouse hippocampus, amygdala, central nucleus, and in the hypothalamic paraventricular nucleus [97].

A recent study assessed the situation of iron in the brain of Tfr2 KO mouse model vs WT sib pairs subjected to an iron-enriched diet (IED). They both are iron overloaded animals, so one could distinguish the effects of Tfr2 silencing from those due to Tfr2-independent iron load modifications.

It has been demonstrated that Tfr2 causes a lack of brain Hepc response to the systemic rise of iron levels, with altered iron mobilization and/or cellular distribution in the nervous tissue [98].

Moreover, Tfr2 KO mice present a selective over activation of neurons in the limbic circuit and the emergence of an anxious-like behavior.

Also, microglial cells showed sensitivity to iron perturbations of Tfr2 KO mice, being more reactive, dystrophic, and with a high level of apoptosis [97]. In light of these data, Tfr2 appears to be a key regulator of brain iron homeostasis, and could have a role in the regulation of the brain regions that are involved in the anxiety onset, mainly, the basolateral and central nucleus subregions of the amygdala [98].

## 5. Tfr2 in Intracellular Iron Trafficking

It is still under debate if Tfr2α contributes to iron introduction inside the cells. When the protein was characterized, it was reported that, in vitro, it was able to introduce iron inside cells [2], but its contribution to intracellular iron amount in vivo seems to be quite negligible, since Tfr1-deficient mice present severe iron deficiency not compensated by the presence of Tfr2 [1].

Conversely, Tfr2α seems to have a role in intracellular iron trafficking, at least in specific cell types. The first evidence about it was found in dopaminergic neurons, where a novel Tf/Tfr2α-mediated iron transport pathway to the mitochondria has been reported [3]. Disruption of this Tf/TfR2α-dependent system has been associated with PD, and this finding highlights the role of iron accumulation in this movement disorder [3]. In this regard, a protective association between some Tf and TfR2α genetic haplotypes and PD was reported, suggesting that Tf or a Tf/TfR2α complex may play a role in the etiology of these disorders [99].

**Figure 4:**
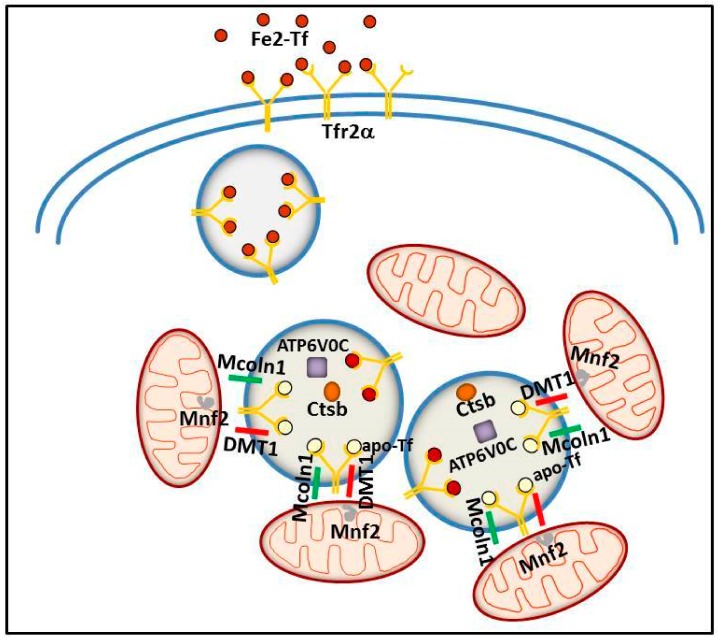
Schematic model illustrating the putative intracellular Tfr2α role in erythroid cells. Tfr2α—together with ATPV0C, a V-ATPase that contributes to vesicular acidification and lysosomal activity; Mcoln1, a lysosomal divalent cation channel; lysosomal cathepsin B (CTSB); and iron importer DMT1—could be involved in iron delivery from lysosome to mitochondria, with the collaboration of Mitofusin-2 (MFN-2), a mitochondrial outer membrane protein involved in mitochondria–endoplasmic reticulum contacts.

More recently, a similar TfR2α function in iron delivery to mitochondria has been convincingly demonstrated in erythroid cells. In an intermediate stage of human erythroid cell maturation, Tfr2α was present in cytoplasmic multi-organellar complexes, formed by lysosomes surrounded by mitochondria, and found to be co-regulated with several proteins, among which, ionic channels and proteins involved in lysosomal modification and in mitochondrial membrane contacts to other intracellular organelles [100]. Therefore, Tfr2α in lysosomes has been proposed to be involved in iron delivery from these organelles to mitochondria, for Hb synthesis. Considering the abovementioned evidence about a specific role for Tfr2α in erythropoiesis, this data might represent one of the molecular processes at the basis of Tfr2α erythropoietic function/s (Figure 4).

In light of the above data, Tfr2α involvement in iron delivery to the mitochondria, notably, seems to work at least in the two compartments in which this protein is significantly produced: brain and bone marrow. Although a similar mechanism has been evidenced also in cell lines derived from other organs (HeLa and hepatoma cell lines) [52,101], it remains an open question if this TfR2α function is present also in other cell types.

## 6. Tfr2 in Other Diseases

Being a “regulator of the iron regulator” hepcidin, Tfr2 transcriptional analysis was attempted to unravel if the Tfr2 isoforms could be reliable markers for some disorders in which iron perturbations occur.

### 6.1. Tfr2 in Cancer

Cancer cells need an increased amount of iron for their growth, and iron importers upmodulation confer a selective advantage to these cells.

Since its characterization, the significant transcription of *TFR2* gene appeared evident in BM cancer cells, in particular, erythroid leukemic cells [5], but also in myeloid malignant cells [2], while Tfr2β seemed much more prevalent than the TfR2α isoform in chronic B cells lymphocytic leukemia (B-CLL) cells [102].

Due to high *TFR2* expression in erythroid lineage, and to its functional relationship with the erythropoietin receptor (EpoR) [68], *TFR2* transcription was evaluated in patients with myelodysplastic syndromes (MDS), a hematopoietic disorder with a variable risk to evolve in acute myeloid leukemia (AML), and in which chronic anemia can be corrected by Epo injection [103]. It has been found that Tfr2α and Tfr2β isoforms, as well as EPOR transcript, have a lower level of transcription in BM from high risk MDS patients, such as RAEB2, compared to controls and low risk MDS cells [104]. Likewise, AML patients with high level of Tfr2α and Tfr2β present an increased survival [105]. Therefore, Tfr2 isoforms might represent good predictive markers for MDS/AML prognosis.

Calzolari et al. 2007 [106] demonstrated *TFR2* expression in colon and ovarian cancer cell lines, as well as in lymphoma and glioblastoma (GBM). Moreover, in glioblastoma TB10 cell line under hypoxic condition, a marked increase of *TFR2* transcription was observed. In these cell lines, *TFR2* high expression is probably correlated with cell proliferation, since Tfr2 silencing inhibited GMB cell growth. Surprisingly, tumor cells from GBM patients with high *TFR2* transcriptional levels present a better prognosis compared to patients with low transcripts. Although, this is probably due to the fact that Tfr2-expressing cells have a highly increased proliferation, so they are more sensitive to temozolomide, the anti-proliferative drug used in GBM therapy, more than to a direct involvement of Tfr2 in the disease course [107].

### 6.2. Tfr2 in Alzheimer’s Disease (AD)

Alzheimer’s disease (AD) is another common degenerative disorder in which iron perturbation has been demonstrated [108].

A recent genetic study from 116 AD patients has found that a Tfr2 single nucleotide polymorphism (rs 7385804) and a Tfr2 haplotype, composed by two SNPs (rs 7385804 and rs 4434553) are associated with a decreased AD susceptibility [109].

In the same study, a significant decrease of Tfr2 transcription was found in peripheral blood mononuclear cells (PBMC) from AD patients, compared to healthy controls (*p* < 0.001) [110].

## 7. Tfr2 as a Therapeutic Target

In our opinion, three might be the major application of Tfr2α as a therapeutic target: (a) as Hepc regulator, it could be a target in disorders in which Hepc amount is, in some way, inadequate to body iron availability; (b) since Tfr2α production is selective in specific organs and cell types, it could represent a selective target to correct iron perturbation in these organs; (c) Tfr2, being a membrane protein that is able to bind plasma Tf and to be internalized with it, this property could be utilized as a vector for drugs.

As mentioned above, TfR2α is involved in regulation of Hepc and, in consequence, in iron modulation according to body/organs needs. Among the Hepc-related disorders there are all the hereditary hemochromatosis forms (Table 1) and the secondary iron overload disorders, like hemoglobinopathy, where abnormally low Hepc amounts lead to iron overload.

In this regard, just published data demonstrate that Tfr2 KO BM transplantation in beta-thalassemia intermedia (β-TI) mouse models significantly improved these animals’ erythropoiesis, opening a new way to the therapy of this very common disorder [111].

In IRIDA and anemia of chronic disease (ACD), where abnormally high Hepc causes the onset of an iron deficiency condition [111], Tfr2 downmodulation might be beneficial to decrease Hepc hyperproduction. A preliminary study on mice revealed that Tfr2 silencing, through small interfering RNAs (Tfr2-siRNA) in a single dose, led to a significant Hepc downmodulation, and increased transferrin saturation within 24 h post-administration, persisting for more than two weeks, and to a recovery from anemia in animal models of ACD [112].

Beside the liver, in brain, Tfr2 regulates the production of local Hepc and iron amount in the CNS, since Tfr2 KO mice brain have a blunted Hepc response to brain iron overload [97]. In recent time, it has emerged that brain Hepc production is altered in several neurodegenerative disorders: downmodulated in Alzheimer’s and Parkinson’s disease [113,114] and upmodulated in restless legs syndrome (RLS) [115]. It might be worthwhile to further investigate if Tfr2 is involved in these Hepc variations, and consider using anti Tfr2 antibodies or siRNA-based therapy to rescue Hepc physiologic values in RLS. Nowadays, siRNA delivery to brain is quite difficult, due to the presence of the BBB, but the ongoing studies on nanoparticles’ use, to target siRNA in specific sites, could allow an increase in the efficacy of this therapy [116].

An alternative therapeutic approach aims to deliver blocking antibody in the brain, exploiting the BBB physiologic activities. This is based on the use of anti-Tfr1 antibodies, since Tfr1 is well expressed in BBB endothelial cells and is involved in receptor-mediated transcytosis. Indeed, it has been demonstrated that the bispecific Tfr1/BACE1 (β-amyloid cleaving enzyme-1) antibody resulted in being effective in decreasing β-amyloid concentration in the brain [117].

Due to high Tfr1/Tfr2 homology and the common cellular internalization through a receptor-mediated endocytosis pathway, one could hypothesize a parallel Tfr2-based approach for CNS, blocking antibody delivery. Nevertheless, it should be further confirmed, the presence of Tfr2 on BBB endothelial cells and its involvement in transcytosis.

Recent studies have aimed at exploiting the Tfr1 as a vehicle for drug delivery inside the cells through endocytosis, often utilizing Tfr1 natural ligand, Tf, conjugated with different synthetic molecules [1]. Moreover, since Tfr1 is able to bind and internalize FtH also [118], FtH nanocages conjugated with a PARP inhibitor, olaparib, were developed for breast cancer therapy [119].

Due to its strict homology to Tfr1, one might hypothesize that Tfr2α could also be utilized to deliver drugs inside cells. This approach would be particularly useful for two main Tfr2α features; Tfr2α-selective expression in particular tissues (hepatic, erythroid, and in CNS) and Tfr2α high expression in several tumor cells, sometimes with Tfr1 and sometimes without it. High expression of Tfr1 and Tfr2α were, in fact, detected in tumor and para-cancerous normal liver tissues collected from 41 patients with hepatocellular carcinoma (HCC) [120]. Tfr2α is also highly expressed in brain tumor cells in several cases of anaplastic astrocytoma and glioblastoma, but not in normal brain or endothelial brain cells [121].

Furthermore, in the light of the new data on Tfr2α function in delivery iron to mitochondria, Tfr2α could, possibly, represent a good vehicle for drug delivery in these organelles [122], paving the way to tailored therapies for mitochondrial iron disorders, notably Friedreich’s ataxia [123].

Unfortunately, too few functional data are available at the moment on Tfr2β isoform to foresee possible therapeutic applications.

## 8. Conclusions

The process of body iron homeostasis is complex, and since there is no apparent significant excretory pathway, the amount of iron absorbed from mature duodenal enterocytes and that is recycled by macrophages needs to be tightly regulated. From the last decades, iron pathways have been enriched of new regulatory proteins whose functions remains to be elucidated from the molecular point of view. Each of them could represent a potential target for a focused pharmacological therapy of disorders with iron unbalance, that still represents the most prevalent of diseases all around the world.

## Figures and Tables

**Table 1:** Hereditary hemochromatosis (HHC types) and their relationship with hepcidin.

HH type	Acronym	Inheritance	Gene	Protein	Function
HFE1	HFE-HHC	AR	HFE	Hfe	Hepc regulator
HFE2a	HJV-HHC	AR	HJV	Hemojuvelin	Hepc regulator
HFE2b	HEPC-HHC	AR	HAMP	Hepc	Fe absorption inhibitor
HFE3	TFR2-HHC	AR	TFR2	Tfr2	Hepc regulator
HFE4	FPN1-HHC	AD	SLC40A1	Fpn1	Hepc receptor
